# Polyester Microfibers Exposure Modulates *Mytilus galloprovincialis* Hemolymph Microbiome

**DOI:** 10.3390/ijms25158049

**Published:** 2024-07-24

**Authors:** Manon Auguste, Martina Leonessi, Lapo Doni, Caterina Oliveri, Anita Jemec Kokalj, Damjana Drobne, Luigi Vezzulli, Laura Canesi

**Affiliations:** 1 Department of Earth, Environment and Life Sciences (DISTAV), University of Genoa, 16132 Genoa, Italy; martina.leonessi@edu.unige.it (M.L.); lapo.doni@unige.it (L.D.); caterina.oliveri@unige.it (C.O.); luigi.vezzulli@unige.it (L.V.); laura.canesi@unige.it (L.C.); 2 NBFC, National Biodiversity Future Center, 90133 Palermo, Italy; 3 Department of Biology, Biotechnical Faculty, University of Ljubljana, 1000 Ljubljana, Slovenia; anita.jemec@bf.uni-lj.si (A.J.K.); damjana.drobne@bf.uni-lj.si (D.D.)

**Keywords:** microfibers, bivalve, microbiota, hemolymph, immunity, inflammation

## Abstract

Microplastic (MP) contamination in the aquatic environment is a cause of concern worldwide since MP can be taken up by different organisms, altering different biological functions. In particular, evidence is accumulating that MP can affect the relationship between the host and its associated microbial communities (the microbiome), with potentially negative health consequences. Synthetic microfibers (MFs) represent one of the main MPs in the marine environment, which can be accumulated by filter-feeding invertebrates, such as bivalves, with consequent negative effects and transfer through the food chain. In the mussel *Mytilus galloprovincialis*, polyethylene terephthalate (PET) MFs, with a size distribution resembling that of an MF released from textile washing, have been previously shown to induce multiple stress responses. In this work, in the same experimental conditions, the effects of exposure to PET-MF (96 h, 10, and 100 μg/L) on mussel hemolymph microbiome were evaluated by 16S rRNA gene amplification and sequencing. The results show that PET-MF affects the composition of bacterial communities at the phylum, family and genus level, with stronger effects at the lowest concentration tested. The relationship between MF-induced changes in hemolymph microbial communities and responses observed at the whole organism level are discussed.

## 1. Introduction

Microplastics (MPs) (from 0.1 to 1000 μm) are ubiquitous contaminants that represent a serious cause for concern due to their potential effects on the environment. MPs found in water compartments show a large variety in terms of composition, size, shape and concentration [1]. In particular, plastic microfibers (MFs) released from synthetic fabrics throughout diverse lifecycle steps, including textile laundering, have been identified as a main source of MP pollution (reviewed in [2]). In samples collected in different world seawater compartments, fibres were almost constantly present, and polyester was the most abundant synthetic polymer [3]. MFs are comprised in the size range from µm up to a few mm and are readily available for some marine fauna.

Marine bivalves (oysters, mussels, clams) are among the foundation species, that is, species representing the biotic basis for many coastal ecosystems worldwide [4], and also represent an important food source and economic value, with a world production of over 15 million tonnes per year [5,6]. Aquacultured and natural populations of bivalves are strongly affected by biotic and abiotic factors: increases in mortality episodes have been attributed to multiple causes, mainly including the involvement of pathogens, but also ocean warming and exposure to contaminants [7,8].

Increasing evidence supports the hypothesis that in bivalve molluscs, as in other organisms, the microbiome (i.e., the microbial community associated with the host) plays an essential role in health and disease, providing benefits for survival, homeostasis, and development [9]. The bacterial component of the bivalve microbiome is known to be involved in a range of functions, including digestion, nutrient cycling, and immune defence, specific to the tissues and location [10,11]. In particular, hemolymph (the circulating fluid), due to the bivalve open circulatory system, is at the interface between the external environment and all tissues and consequently shows a high bacterial diversity, with strong interactions between microorganisms and immune effectors [12].

A recent FAO report summarised current knowledge on the impact of different types of MPs on the host gut microbiome, from aquatic organisms to humans: in general, MP exposure led to changes in the microbiota diversity and composition, with effects depending on the type of polymer, particle size and shape, and pristine/weathered particles [13]. However, only a few studies investigated other biological effects on the host and their relationship with changes in the microbiome. In particular, at present it cannot be understood whether the gut microbiome enhances the impact of MPs on the host, or if microbial dysbiosis is the consequence of the host response to MPs [14]. Establishing causality and the underlying mechanisms of the host–microbiome interactions would contribute to better assessing the impact and risk posed by MPs. Most studies utilised aquatic organisms as experimental models, including species of commercial importance as seafood for humans: among these, bivalve molluscs are of particular interest because, as filter feeders, they can concentrate different contaminants, including MPs, and they are consumed whole, acting as potential vehicles for MPs. Several studies reported the presence of MPs, including MFs, in mussels, due to different mechanisms, such as ingestion, adherence, and fusion into the byssus (reviewed by [15]). Moreover, previous data have shown that exposure to different types of microplastics and nanoplastics can affect the mussel microbiome in the hemolymph, gut, and whole animals, respectively [13,16,17,18]. The results suggest that alteration of the associated microbial communities may represent a common effect of MP contamination of edible bivalves.

The impact of polyethylene terephthalate PET-MF, one type of polyester polymer, has been recently investigated in *Mytilus galloprovincialis* [19]. MFs obtained by cryo-milling of a fleece cover were utilised [20], yielding fibres of various dimensions, resembling those produced after the washing machine cycle [21], and including those that can be ingested by mussels due to particle selection through the gill filtering activity [22]. The results demonstrated that in vivo exposure to PET-MF has a significant impact on mussel physiology at environmental exposure levels, affecting multiple processes in different tissues [19]. In particular, data obtained in the hemolymph showed that MFs stimulated extracellular immune responses, indicating induction of immune/inflammatory processes.

In the present work, the possible effects of exposure of *M. galloprovincialis* to PET-MF in the same experimental conditions (96 h, 10, and 100 μg/L, corresponding to about 150 and 1500 MF/mussel/L, respectively) on hemolymph microbiota composition were evaluated by 16S rRNA gene amplification and sequencing. The results are discussed in light of other effects previously observed at multiple levels.

## 2. Results

### 2.1. MF-Characteristics

The MFs used in the present work were previously characterised [19,20], and data on MF characterisation in terms of appearance and size distribution are summarised in Figure S1. The MFs used in the present work are of a pink colour (Figure S1A), with an average length of 228.6 ± 185.5 µm, width of 28.3 ± 6.7 µm, and a large size range distribution (Figure S1B). Moreover, the polymer composition was reported as polyethylene terephthalate (PET) by µ-Raman spectroscopy [19].

### 2.2. Microbiota Profile

The microbial composition of hemolymph of *Mytilus* was evaluated and a total of 2136 species were identified for all samples (for each group of samples an average of C: 656 features, F1: 662 features, and F2: 1570 features).

The microbial profile in the hemolymph of *M. galloprovincialis* in control samples and samples exposed to PET-MF at both 10 and 100 µg/L for 96 h (F1 and F2, respectively) was analysed at different levels of the phylogeny.

**Figure 1:**
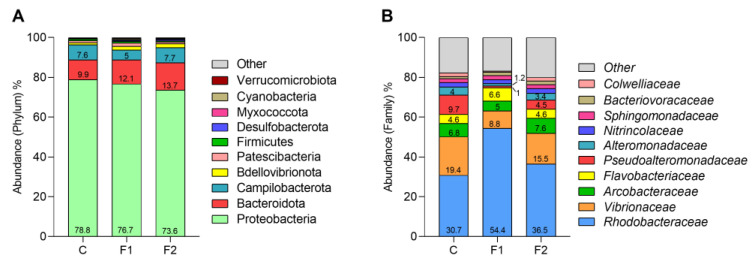
Variations in the relative abundance of microbial communities in *M. galloprovincialis* hemolymph after 96 h exposure to PET-MF at the phylum level (**A**) and at the family level (**B**). The top 10 taxa are reported. C: control, F1: 10 µg/L, and F2: 100 µg/L.

In all hemolymph samples, the microbial community was dominated by three main phyla: Proteobacteria (about 76.4%), Bacteroidota (11.9%), and Campilobacterota (6.8%), followed by lower proportions of Bdellovibrionota (1.56%), Patescibacteria (1.28%), and Firmicutes (0.71%). All other phyla were present at abundances <0.5% (Figure 1A and Table S1).

In MF-exposed mussels, a progressive decrease in Proteobacteria (from 79% in Controls to 73.6% in F2 samples, (*p* ≤ 0.05)) and an increase in Bacteroidota (from 9.9 to 13.7%, *p* ≤ 0.05) were observed. In F1 samples, the abundance of Campylobacterota was also decreased (from 7.6 to 5%). Moreover, among less represented phyla (<1%), increases in abundance were observed irrespective of the MF concentration: about 2-fold in Bdellovibrionota and Desulfobacterota, 2–4-fold in Cyanobacteria, and 6-fold in Verrucomicrobiota. Only Firmicutes showed a decrease in F2 samples (about 30%; *p* ≤ 0.05).

Exposure of mussels to PET-MF induced a more evident shift in microbiota composition at the family level. The top 10 families accounted for 80% of the total microbial community (Figure 1B and Table S2). *Rhodobacteraceae* and *Vibrionaceae* were the most represented families in all groups. The abundance of *Rhodobacteraceae* was increased in MF-exposed groups, with stronger effects in F1 samples (from ~30.7 in controls to 54.4% in F1 and 36.5% in F2, corresponding to +77% and +19%, respectively, *p* ≤ 0.05 for both groups). On the contrary, *Vibrionaceae* showed lower abundances, with a drop from 19.4% in controls to 8.7% in the F1 group (*p* ≤ 0.05) and 15.4% in F2 (−55% and −21%, respectively). MF exposure also greatly reduced the abundance of *Pseudoalteromonadaceae* with respect to controls in F2 (from 9.7% to 4.5%); in particular, a dramatic decline was observed in F1 (0.9%, *p* ≤ 0.05). At this concentration, a similar trend was observed for *Alteromonadaceae*, with F1 samples showing a large decrease (from 4 to 1.17%, corresponding to −70% of controls, *p* ≤ 0.05 for both groups).

At the genus level, the overall microbiome composition of mussel hemolymph was dominated by 12 genera that accounted for >50% of the total abundance (Table S3). Among these 12 genera, 8 belong to the *Rhodobacteraceae* family (relative abundance from 21 to 36%). *Vibrio* spp. represented the second most abundant genus in all samples (average ~14%) followed by *Pseudoalteromonas* spp. (~5%). A significant proportion of genera belonging to *Arcobacteraceae-unc* spp. (~4%) were also identified. Table S3 also reports data obtained from control and MF-exposed mussels.

**Figure 2:**
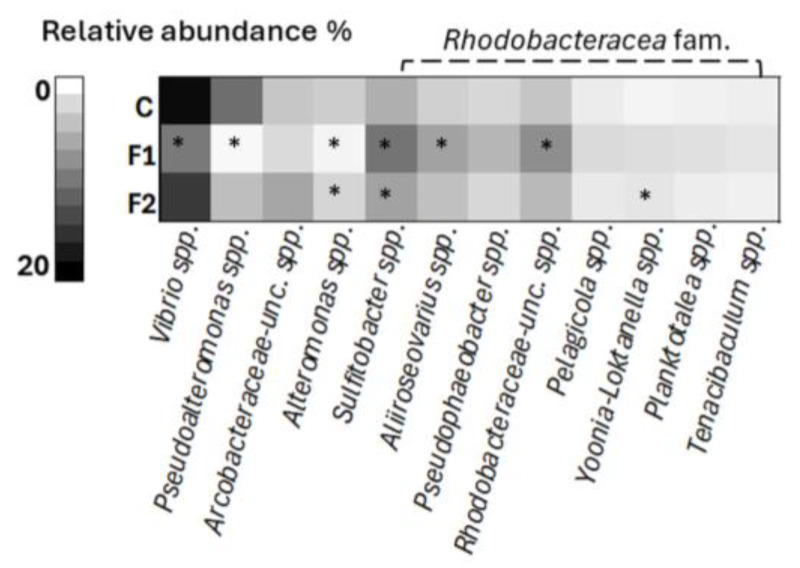
Heatmap analysis of the microbial community at the genus level of *M. galloprovincialis* hemolymph. Data represent the top 12 taxa with abundance >1% of the total. Data are the mean of two individual samples for each condition, C: control, F1: 10 µg/L, F2: 100 µg/L, and * *p* ≤ 0.05 with respect to control (non-parametric Kruskal–Wallis test followed by post hoc Dunn’s test (Bonferroni adjusted)).

The overall changes induced by MF exposure are clearly depicted in a heatmap of individual genera with abundance ≥1% (Figure 2). *Vibrio* spp. abundance was halved in the F1 group (from 19% in controls to 8.7% in F1, *p* ≤ 0.05), whereas a smaller decrease was observed in F2 (14.7%). A dramatic decline was observed in F1 for *Pseudoalteromonas* spp. (from 9.7% in controls to 0.9%, *p* ≤ 0.05) that was again smaller in F2 (6%). In contrast, an increase in *Arcobacteraceae-unc* spp. was observed in F2 (5.8% vs. 3.9% in C). Moreover, Figure 2 emphasises the increase in abundance of several genera belonging to *Rhodobacteraceae* in samples of the F1 group, such as *Sulfitobacter* spp. (9.2% in F1 vs. 5.2% in C, *p* ≤ 0.05), *Aliiroseovarius* spp. (6.1% in F1 vs. 3.3% in C, *p* ≤ 0.05), and *Pseudophaeobacter* spp. (4.8% in F1 vs. 2.9% in C). Smaller changes were observed in F2 samples.

Among the genus *Vibrio* spp., the method used for sequencing also allowed for the identification at the species level. As previously described in *Mytilus* spp. [23], we mainly detected species belonging to the *Vibrio splendidus* clade, which includes pathogens of bivalves (see Table S4 for details).

**Figure 3:**
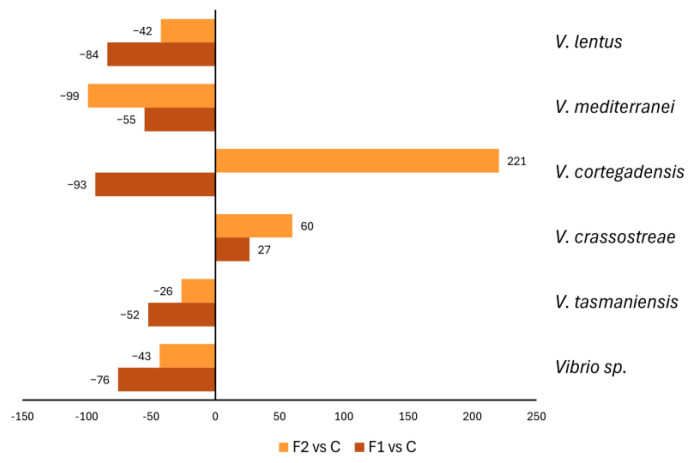
Changes in abundance of the main *Vibrio* species expressed as % variation (>to 50%) in hemolymph samples of MF-treated mussels with respect to control (unexposed) samples. Data are the mean of two individual samples for each condition, C: control, F1: 10 µg/L, and F2: 100 µg/L.

The different *Vibrio* species identified in the microbiota of the F1 and F2 groups were normalised as % of control samples, and those showing larger variations in abundance (cutoff ±50%) are shown in Figure 3. MF exposure induced a general decrease in most Vibrio species, in particular in F1 samples, that was highest for *V. lentus* (−84%), *Vibrio* sp. (−75%), *V. mediterranei*, and *V. tasmaniensis* (−55 and −52%, respectively). In contrast, some *Vibrio* species were increased after exposure to MF: in F2, +220% for *V. cortegadensis*, and *V. crassostreae* in both exposure groups (+26% in F1 and +60% in F2). Interestingly, some species, such as *V. tapetis*, were not identified in the controls but only after exposure to both concentrations of MF (Table S4).

Among species belonging to the Rhodobacteracea family, the most abundant were Pelagicola litoralis, Aliiroseovarius halocynthiae, Sulfitobacter pseudonitzschiae, Planktotalea lamellibrachiae, Sulfitobacter geojensis, and Aliiroseovarius sediminilitoris (>0.5% abundance) (Table S5). The largest increases in abundance were observed in F1 samples, in particular for Ruegeria conchae, Sulfitobacter mediterraneus, and Octadecabacter temperatus (from <0.03% for all species in controls to 0.23%; 0.19% and 0.18% in F1, respectively; corresponding to increase of >200% with respect to controls).

**Figure 4:**
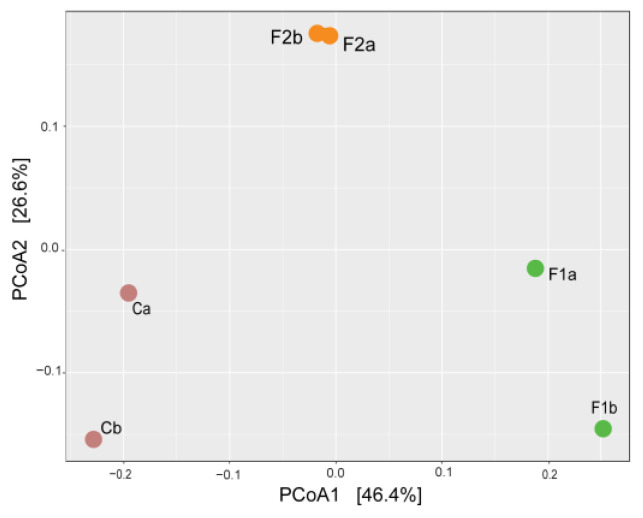
Principal coordinate analysis (PCoA) plot with Blay–Curtis dissimilarity. PCoA plots of beta diversity for each replicate sample and the explained variations are explained in brackets. C: control, F1: 10 µg/L, and F2: 100 µg/L. Letters a and b indicate two individual samples, each obtained by the pooled hemolymph from 10 mussels.

Finally, in order to visualise the overall data, a principal coordinate analysis (PCoA) plot was generated (Figure 4), based on Bray–Curtis beta-diversity with two components, providing a measure of the differences in community composition based on ASVs (amplicon sequence variants), regardless of taxonomic assignment. PCoA1, which accounts for 46.4% of the variation, clearly separates control samples (Ca and Cb) from MF-exposed samples, with F1a and F1b being more distant from controls than F2a and F2b. It is worth noting that F2 samples were also separated according to PCoA2 (26.6% of total variance), further illustrating the distance among groups.

## 3. Discussion

We have recently shown that exposure of *M. galloprovincialis* to PET-MF (96 h, 10, and 100 μg/L) induced a variety of physiological responses [19]. In the present work, the effects of MF, in the same experimental conditions, were further investigated to assess the impact on the hemolymph microbiota: this compartment was selected in order to investigate the possible relationship between the host immune response and its microbiome, in analogy with previous studies with nanoplastics and other nanoparticles [16,24]. The results obtained show that PET-MF induced a shift in microbiota composition at different taxonomic levels.

In control mussels the hemolymph bacterial community was dominated by the phyla Proteobacteria > Bacterioidota > Campylobacteriota (>90%); the most represented families were *Rhodobacteraceae*, *Vibrionaceae*, *Flavobacteriaceae*, and *Arcobacteraceae*. The present data, obtained in mussels sampled in June from a farm in La Spezia (IT), are in line with those previously observed in May-July in mussels from the same farming site [25], and therefore within the seasonal variations of hemolymph microbiome for that mussel population.

In both MF exposure groups (F1 and F2), PET-MF induced changes in abundance of the three most represented phyla, with a decrease in Proteobacteria and Campylobacteriota and an increase in Bacterioidota. Changes in Proteobacteria and Bacterioidota represent a common effect of MP exposure on the microbiome of different animal models [26]. Increases in several of the less-represented phyla were also observed. Moreover, in samples exposed to the highest MF concentration (F2), the Firmicutes/Bacteroidetes (F/B) ratio was decreased (from 0.074 to 0.042), as similarly observed in the gut microbiota of mussels exposed to PET-MF [18].

In humans, this parameter has been broadly associated with the maintenance of intestinal homeostasis, and its alterations are considered a dysbiosis that can lead to various pathologies [27]; in particular, a decrease in the F/B ratio is associated with inflammatory diseases [28]. Even though Firmicutes are the predominant phyla together with Bacteroidetes in human gut microbiota, but not in bivalves, a change in their ratio may reflect a similar condition also in mussels, but this needs to be investigated further.

A shift in microbiota composition was more clearly observed at the family level, where *Rhodobacteraceae* and *Vibrionaceae* were the most represented families in all groups. Interestingly, only in samples exposed to the lowest MF concentration (F1) was the abundance of *Rhodobacteraceae* further increased, whereas that of *Vibrionaceae* was decreased. *Rhodobacteraceae*, comprising about 170 genera, are among the most widely distributed bacterial lineages in marine habitats [29] and represent significant components of bivalve microbiota, which are considered beneficial to the host, contributing to reducing pathogen load and improving immune response [30]. Their metabolism in fact includes the utilisation of inorganic and organic compounds, sulphur oxidation, aerobic anoxygenic photosynthesis, carbon monoxide oxidation and production of secondary metabolites, and degradation of oil hydrocarbons [31]. Members of *Rhodobacteraceae* contributed significantly to the gut microbial communities in the mussel *Crenomytilus grayanus* from polluted coastal areas, suggesting a possible role in the detoxication of xenobiotics [32]. Interestingly, *Rhodobacteraceae* have been regularly found associated with plastic debris, it has been hypothesised that some of these bacteria could play a role in the colonisation and possible degradation of plastic in the oceans [33] and may represent putative degraders of low-density polyethylene-derived compounds [34].

In F1 samples, the decrease in *Vibrionaceae* could be partly due to the stimulation of extracellular immune defences (ROS, NO, lysozyme) previously observed in the hemolymph of mussels in the same experimental conditions [19], leading to increased bactericidal activity towards *Vibrio* sp., some members of which represent the main natural bacterial pathogens for bivalves [35].

MF exposure also reduced the abundance of both *Pseudoalteromonadaceae* and *Alteromonadaceae*, in particular at the lowest concentration tested. Members of these families are considered beneficial for bivalve growth and development, inhibition of adherence and colonisation of pathogenic bacteria, modulation of the gut microbiota, and immune response ([30] and refs. therein).

The effects of lower concentrations of MFs were further confirmed at the genus level, where MF increased the abundance of genera belonging to *Rhodobacteraceae*, in particular *Sulfitobacter* spp., *Aliiroseovarius* spp., and *Pseudophaeobacter* spp., and decreased that of *Vibrio* spp. and *Pseudoalteromonas* spp. Interestingly, similar results were observed in the hemolymph of mussels exposed to TiO_2_ nanoparticles, where a decrease in abundance of the genus *Vibrio* was associated with the stimulation of immune defences [24]. Finally, for the genus *Vibrio*, changes at the species level were identified. MF exposure, again at the lowest concentration, resulted in large decreases of several *Vibrio* species, including potential bivalve pathogens. Overall, the observed changes in vibrio abundance thus suggest a specific immunostimulation towards potential pathogens.

To date, few studies are available on the effects of different plastic MFs on mussel responses and gut microbiome. Exposure to low concentrations (50 and 100 particles/L) of nylon MFs for 21 days did not affect gut microbial communities nor resulted in tissue damage [36]. In contrast, exposure to high concentrations (>1 mg/L) of PET-MF of different sizes affected the stomach microbiome [18]. In particular, in samples exposed for 4 days to small-size MFs (representing the experimental conditions closer to those here utilised), changes in bacterial composition at the phylum level were similar to those observed in the present work (i.e., decrease in Proteobacteria, increase in Bacteroidota, change in the F/B ratio). Overall, our results demonstrate that exposure to PET-MF, in a mixture of various sizes, resembling those released after textile washing, in particular at low, environmentally realistic concentrations (10 µg/L, 150 particles/L), induced a shift in the bacterial communities associated with mussel hemolymph. PCoA analysis confirmed the strong separation of F1 samples with respect to both control and F2 samples.

The impact of MPs on host microbiota and health and disease has been investigated in several vertebrate and a few invertebrate models [13]. However, most studies have been carried out on gut microbiome: in general, exposure to MPs altered gut bacterial diversity resulting in loss of commensals and increases in pathobionts (leading to so-called dysbiosis), and caused other negative effects, including changes in gut metabolic profiles and inflammation (reviewed by [26,37]). Although different links have been proposed between MP ingestion, dysbiosis of the gut microbiome, and health effects, common patterns of perturbation of animal microbiomes by different stressors leading from a healthy to a dysbiotic stable state are difficult to identify [38]. In this light, changes in microbiome components induced by different MPs, in different experimental conditions, model organisms, and body compartments, cannot be easily compared.

In particular, in invertebrate models, studies on hemolymph microbiome still represent a minor proportion of published papers [26]. However, in the bivalve open circulatory system, hemolymph is not only in direct contact with all tissues, but it also reflects exchanges with the surrounding environment, thus representing a general indicator of the potential changes in the overall bacterial communities associated with the bivalve host.

When we look at perturbations of the hemolymph microbiome (this work) and the different types of effects induced by PET MF observed in mussels in the same experimental conditions [19] some relationship can be observed. After 96 h of exposure, MF exposure resulted in multiple stress responses at the cellular and tissue levels, including immune stimulation and systemic inflammation (details reported in Table S6). MFs induced lysosomal stress in the hemocytes at both concentrations, but stimulation of hemolymph extracellular immune defences, corresponding to an inflammatory response at the systemic level, was observed only in F1 samples. Increases in activities of the antioxidant enzymes CAT and GST, indicating oxidative stress, were also observed in both gills and digestive gland in F1, but not in F2 samples. MFs induced histopathological changes in both tissues, including hemocytic infiltration, indicating tissue inflammatory processes, independent of concentration; however, an increase in mucus production in digestive tubules was observed only in F1 samples [19]. The amount of MF retained by the tissues (gills and digestive gland) was extremely low with respect to the nominal exposure concentration, in line with the knowledge that >90% of anthropogenic particles are egested by bivalves within 48 h [22]; however, the amount of MF detected in tissues (with respect to nominal exposure concentrations) was higher in mussels exposed to the lower, environmentally relevant concentration [19].

The results here obtained show that, in these conditions, the shift in bacterial community composition induced by MFs includes increases in taxonomic groups that are considered beneficial for the bivalve host (i.e., Rhodobacteria) and decreases in others, including potential pathogens, such as Vibrios. In this light, the observed changes in hemolymph microbiota may contribute to the maintenance of homeostatic processes during transient stressful conditions induced by MF exposure.

## 4. Materials and Methods

### 4.1. Microfiber Characteristics

The polyethylene terephthalate (PET) microfiber-MFs used in this study were previously characterised in [19,20]. The MFs used in the present study were obtained after cryo-milling of a pink polyester fleece blanket, and fully characterised using different instruments µ-Raman spectroscopy (for polymer composition), SEM, and light microscopy (for surface details and measurement of size range fragments) (for further details see methods section in [19]).

### 4.2. Animals and Treatments

Mussels (*Mytilus galloprovincialis* Lam.) were purchased in June 2022 from an aquaculture farm in the Ligurian Sea (La Spezia, Italy) and were transferred to the laboratory and acclimatised in static tanks containing aerated artificial seawater (ASW [39], pH 7.9–8.1, 35‰ salinity (1 L/animal)) at 18 °C for 24 h prior the exposure.

Animals (two tanks per condition containing 10 mussels each) were exposed for 96 h to MFs at concentrations of 10 μg/L and 100 μg/L, corresponding to about 150 and 1500 fibers/L/mussel, as previously described [19]. MF suspensions in ASW (2 mg/mL) were spiked daily into experimental tanks (1 L/mussel, total of 10 L per tank) to reach the desired concentration. ASW was changed each day and MFs were immediately added after ASW renovation. Parallel groups of control (non-exposed) mussels were kept in clean ASW (two replicate tanks of 10 mussels each). Animals were not fed during the experiments. No mortality was observed in different experimental conditions. At the end of the exposure, hemolymph was extracted from the adductor muscle of animals of each condition, using a sterile syringe (18 G1/2″ needle), filtered with gauze, and pooled (two pools of 10 animals each) at 18 °C.

### 4.3. Preparation of DNA Library and Sequencing

Microbial DNA was extracted from an aliquot of 500 µL of whole hemolymph of control and MF-exposed (10 and 100 µg/L) mussels using DNeasy Blood and Tissue kit from Qiagen according to the manufacturer’s instructions (Qiagen, Hilden, Germany). The amount of DNA extracted was determined fluorometrically with the Quantifluor dsDNA system (Promega Italia srl, Milano, Italy).

We obtained 16S rRNA PCR amplicon libraries using the 16S barcoding kit 1–24 (SQK-16S024) from Oxford Nanopore Technologies, using the primers 27F and 1492R (cover nearly full-length of 16S rRNA gene) and following the manufacturer’s protocol. All six barcoded libraries were pooled (10 ng of DNA) and loaded on a MinION flow cell 9.4.1 (flow cell priming kit, EXP-FLP002).

### 4.4. Bioinformatic Analysis

Bioinformatic analysis was performed using a similar pipeline for Nanopore sequencing data as that reported by Latorre-Pérez et al. (2021) [40]. This analysis is based on the use of the Spaghetti bioinformatic pipeline for exploratory analysis and data visualisation. The pipeline relies on the following steps: Porechop (v. 0.2.4) was used to remove sequencing adapters from reads. The reads (between 1200 and 1800 bp) were filtered using Nanofilt (v. 2.7.1). A quality check was carried out with NanoStat (v. 1.4.0) and the detected chimeras were removed by yacrd. Filtered reads are mapped against the SILVA database (v. 138), as formatted and provided by Qiime2, by using minimap2 (v. 2.17-r9419). Finally, the alignments were filtered using Python scripts (included in the pipeline), and taxonomy and abundance tables were obtained. For details and an explanation of the pipeline steps, refer to Spaghetti’s GitHub repository (https://github.com/adlape95/Spaghetti, accessed on 20 March 2024). All SSU rRNA data were deposited in the NCBI SRA repository (accession number: PRJNA1107368).

### 4.5. Statistical Analysis

The data obtained at different taxonomic levels (for Phylum the 10 most abundant taxa, Family the 10 most abundant, Genus 12 most abundant taxa) and for each dataset control vs. each MF treatment were analysed by a non-parametric Kruskal–Wallis test followed by post hoc Dunn’s test (Bonferroni adjusted) (*p* ≤ 0.05). Statistical analyses were performed using R V4.3.3, “Angel Food Cake” (R Core Team, 2024) in RStudio Server V2023.09.1 “Desert Sunflower” (R Studio Team 2023).

## 5. Conclusions

**Figure 5:**
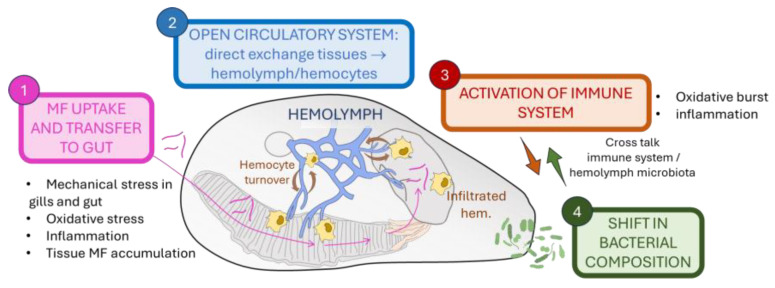
Tentative model summarising the possible serial effects of MF uptake by mussels and transfer into the body, including the mechanisms that could lead to a shift in microbiota in the hemolymph. The figure gathers the results obtained in the present work and in Auguste et al. (2023) [19].

In light of the results obtained, in analogy with the conceptual model of the possible impact points of MP ingestion on the gut microbiome and the mechanisms leading to gut dysbiosis [37], a tentative scheme summarising the possible consequences of MF ingestion by mussels which could lead to changes in hemolymph microbiome can be drawn (see Figure 5). (1) MFs can cause mechanical stress during uptake by the gills and transfer through the gut, leading to tissue oxidative stress, inflammation, and hemocytic infiltration, this can directly lead to changes in the gut microbiome, as described by Park et al. (2024) [18], (2) Due to the continuous interchange between hemolymph and tissues, this can induce stress in circulating hemocytes and release of antimicrobial factors (ROS, NO, lysozyme). (3) Activation of the immune system may indirectly induce changes in the hemolymph microbiome. Modulation of the cross-talk between the immune system and hemolymph microbiota may result in either shifts in microbiome composition that contribute to homeostasis maintenance during MF exposure or trigger the onset of diseases, deteriorate host health, and promote pathogenic infection.

However, as recently underlined in the FAO report on MP and the microbiome, among research gaps for risk assessment of MP, including MFs, the need emerges to evaluate causality and the mechanistic evidence that confirm if MP-induced dysbiosis is a direct effect of MP on the microbial population, an indirect outcome resulting from the host response to MP, or a combination of both [13]. Overall, the results of the present work contribute to increasing knowledge on the mechanisms of action and possible overall impacts of MFs, one of the most widespread MPs in the ocean, on key species of marine invertebrates.

## Data Availability

The original contributions presented in this study are included in the article/Supplementary Materials, further inquiries can be directed to the corresponding author/s.

## Supplementary Materials

The following supporting information can be downloaded at: https://www.mdpi.com/article/10.3390/ijms25158049/s1.
