# Supplementary material for: Polyester Microfibers Exposure Modulates Mytilus galloprovincialis Hemolymph Microbiome
Source: Int J Mol Sci. 2024 Jul 24;25(15):8049. doi: 10.3390/ijms25158049 (PMC11312190; doi:10.3390/ijms25158049)
Supplement: Supplementary file 1 [file ijms-25-08049-s001.zip › ijms-3093741-supplementary.pdf]

Supplementary material

Polyester microfibers exposure modulates *Mytilus galloprovincialis* hemolymph microbiome

Manon Auguste[1,2,*], Martina Leonessi [1,2], Lapo Doni[1,2], Caterina Oliveri[1], Anita Jemec Kokalj[3], Damjana Drobne[3], Luigi Vezzulli[1,2] and Laura Canesi[1,2]

[1] Dept. of Earth, Environment and Life Sciences (DISTAV), University of Genoa, 16132 Genoa, Italy
[2] NBFC, National Biodiversity Future Center, Palermo 90133, Italy
[3] Dept. of Biology, Biotechnical Faculty, University of Ljubljana, 1000 Ljubljana, Slovenia
* Correspondence: manon.auguste@edu.unige.it

Figure S1. Summary of PET-MF characterization. A) Representative image from light microscopy showing the pink fibers (scale bar 100 µm). B) average length and width of MF and the different size range with minimal and maximal values measured (n=390) (B) (from Auguste et al,. 2023).

A

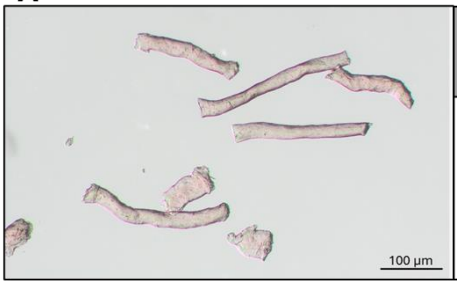

B

PET-MF	Length (µm)	Width (µm)
Mean ± Sd	228.6 ± 185.5	28.3 ± 6.7
Range: Min –Max size	24.4 - 969.1	9.8 - 67.6

Table S1. Bacterial abundance at the phylum level (expressed in %) in the hemolymph of *M. galloprovincialis* after 96 h exposure to PET-MF. C: control, F1: 10 µg/L, F2: 100 µg/L. Letters a and b indicate two individual samples, each obtained by the pooled hemolymph from 10 mussels.

Phylum	Ca	Cb	F1a	F1b	F2a	F2b
Proteobacteria	79.50	78.25	79.57	73.84	74.70	72.54
Bacteroidota	9.73	10.03	10.82	13.29	12.50	14.84
Campilobacterota	7.34	7.81	3.25	6.81	7.55	7.76
Bdellovibrionota	0.91	1.06	1.66	1.88	1.92	1.92
Patescibacteria	1.32	1.04	2.16	1.33	1.02	0.81
Firmicutes	0.59	0.87	0.74	0.89	0.62	0.54
Desulfobacterota	0.09	0.47	0.07	0.82	0.60	0.63
Myxococcota	0.34	0.23	0.46	0.41	0.48	0.32
Cyanobacteria	0	0.20	0.67	0.27	0.28	0.23
Verrucomicrobiota	0.09	0	0.34	0.20	0.25	0.28
Planctomycetota	0	0	0	0.06	0.04	0.06
Dependentiae	0	0	0.13	0	0	0

Acidobacteriota	0	0	0.07	0.03	0.02	0
Actinobacteriota	0.09	0	0	0	0	0.03
Deinococcota	0	0	0	0.10	0	0
Dadabacteria	0	0	0.071	0	0	0.01
Fusobacteriota	0	0	0	0.08	0	0
NB1-j	0	0.04	0	0	0	0
LCP-89	0	0	0	0	0.01	0.02
Fibrobacterota	0	0	0	0	0	0.01
Calditrichota	0	0	0	0	0.01	0

Table S2. Bacterial abundance at the family level (top 10 taxa expressed in %,) in the hemolymph of *M. galloprovincialis* after 96 h exposure to PET-MF. C: control, F1: 10 µg/L, F2: 100 µg/L. Letters a and b indicate two individual samples, each obtained by the pooled hemolymph from 10 mussels.

Family	Ca	Cb	F1a	F1b	F2a	F2b
Rhodobacteraceae	32.26	29.23	57.31	51.46	37.15	35.79
Vibrionaceae	18.56	20.19	8.26	9.26	15.16	15.71
Arcobacteraceae	6.52	6.99	3.25	6.81	7.52	7.69
Flavobacteriaceae	4.39	4.72	6.61	6.51	3.97	5.17
Pseudoalteromonadaceae	9.23	10.18	1.12	0.82	4.89	4.08
Alteromonadaceae	3.85	4.27	1.06	1.29	3.28	3.52
Nitrincolaceae	2.23	2.16	2.22	1.78	2.48	2.36
Sphingomonadaceae	2.35	1.62	2.15	1.86	1.93	1.91
Bacteriovoracaceae	0.91	1.06	1.66	1.88	1.89	1.85
Colwelliaceae	1.86	1.75	0.53	0.51	1.90	1.57
Other	17.85	17.81	15.85	17.82	19.82	20.36

Table S3. Bacterial abundance at the genus level (20 top genera expressed in %) in the hemolymph of *M. galloprovincialis* after 96 h exposure to PET-MF. C: control. F1: 10 µg/L. F2: 100 µg/L. Letters a and b indicate two individual samples, each obtained by the pooled hemolymph from 10 mussels.

Genus	Ca	Cb	F1a	F1b	F2a	F2b
Vibrio	18.11	19.97	8.19	9.26	14.47	14.96
Sulfitobacter	5.58	4.98	10.20	8.15	6.08	6.00
Pseudoalteromonas	9.23	10.18	1.05	0.82	4.73	3.97
Aliiroseovarius	3.10	3.50	6.61	5.49	4.24	4.24
Arcobacteraceae-unc.	3.58	4.19	1.49	3.94	5.80	5.77
Pseudophaeobacter	2.98	2.83	5.10	4.54	3.07	2.77
Rhodobacteraceae-unc.	4.43	3.47	7.36	7.10	4.81	4.48
Alteromonas	3.44	3.58	1.06	1.29	2.94	3.07
Pelagicola	1.60	1.71	2.88	2.60	1.82	1.80
Yoonia-Loktanella	1.43	0.92	2.72	2.38	2.14	1.96
Planktotalea	1.38	1.34	2.65	2.06	1.50	1.66
Tenacibaculum	1.49	1.57	2.24	1.91	1.09	1.68

Poseidonibacter	1.60	1.60	1.76	1.88	1.20	1.30
Ruegeria	1.70	1.31	2.15	1.62	1.21	1.12
Roseovarius	0.76	0.73	1.93	2.16	1.22	1.08
Peredibacter	0.73	0.81	1.52	1.41	1.50	1.45
Amphritea	1.55	1.38	0.65	0.71	1.41	1.35
Octadecabacter	0.70	0.77	1.61	1.36	1.14	1.22
Pontivivens	1.00	1.16	1.26	1.35	1.04	0.97
Colwellia	1.52	1.41	0.53	0.43	1.60	1.27
Other	34.06	32.59	37.05	39.56	36.99	37.87

Table S4. Abundance of *Vibrio* species (expressed in %) in the different treatment groups identified in the hemolymph of *M. galloprovincialis* after 96 h exposure to PET-MF. C: control. F1: 10 µg/L. F2: 100 µg/L. Letters a and b indicate two individual samples, each obtained by the pooled hemolymph from 10 mussels.

Vibrio species	Ca	Cb	F1a	F1b	F2a	F2b
Vibrio sp.	8.14	9.01	1.75	2.43	4.67	5.06
V. alginolyticus	2.49	2.85	2.04	2.31	2.86	2.81
V. tasmaniensis	3.10	3.02	1.37	1.55	2.27	2.23
V. splendidus	2.30	2.51	1.51	1.13	2.37	2.28
V. crassostreae	0.27	0.49	0.43	0.54	0.59	0.63
V. cortegadensis	0.09	0.37	0	0.03	0.68	0.79
V. mediterranei	0.62	0.69	0.44	0.14	0.01	0.00
V. lentus	0.34	0.31	0.00	0.10	0.16	0.21
V. celticus	0.33	0.14	0.07	0.22	0.17	0.14
V. tapetis	0	0	0.25	0.29	0.15	0.23
V. atlanticus	0.18	0.07	0.07	0.09	0.19	0.16
V. cyclitrophicus	0	0.11	0.07	0.19	0.16	0.20
V. gigantis	0	0	0.13	0.12	0.09	0.04
V. toranzoniae	0.18	0.12	0	0.03	0.01	0.02
V. artabrorum	0.09	0.04	0.07	0.03	0.01	0.04
V. gallaecicus	0	0.07	0	0	0.01	0.04
V. chagasii	0	0.07	0	0	0.01	0.02
V. shilonii	0	0.07	0	0	0	0
V. diabolicus	0	0	0	0.03	0	0.04
V. parahaemolyticus	0	0	0	0.03	0.02	0.01
V. harveyi	0	0.04	0	0	0	0
V. campbellii	0	0	0	0	0.01	0.01
V. comitans	0	0	0	0	0	0.01
V. owensii	0	0	0	0	0.01	0

Table S5. Abundance of the most abundant (top 20 taxa) species from the *Rhodobacteracea* family in the different treatment group identified in the hemolymph of *M. galloprovincialis* after 96h exposure to PET-MF. C= control. F1= 10 μg/L. F2= 100 μg/L. Letters a and b indicate two individual samples, each obtained by the pooled hemolymph from 10 mussels.

Species from *Rhodobacteracea* family	Ca	Cb	F1a	F1b	F2a	F2b
Pelagicola litoralis	5.58	5.63	7.02	6.88	5.68	5.63
Aliiroseovarius halocynthiae	3.03	3.63	4.66	4.47	3.97	4.10
Sulfitobacter pseudonitzschiae	3.25	1.96	4.85	4.21	3.32	3.45
Planktotalea lamellibrachiae	2.45	2.62	4.39	4.34	3.46	3.39
Sulfitobacter geojensis	3.10	3.02	3.49	3.74	2.97	2.62
Aliiroseovarius sediminilitoris	2.01	3.28	1.76	3.22	3.60	3.66
Ruegeria atlantica	2.97	2.04	3.57	1.88	2.09	2.27
Roseovarius scapharcae	1.23	1.73	2.65	3.24	2.40	2.08
Sagittula marina	1.48	1.65	2.44	2.35	1.56	1.81
Ruegeria mediterranea	0.92	2.00	2.05	2.54	1.70	1.56
Roseovarius albus	1.88	1.65	1.41	2.56	1.53	1.74
Sulfitobacter pontiacus	1.07	1.06	1.27	1.52	1.58	1.49
Yoonia maricola	0.53	1.38	1.68	1.89	1.22	1.02
Cognatishimia maritima	1.48	1.65	1.07	1.61	1.00	0.96
Octadecabacter ascidiaceicola	0.92	1.17	1.68	1.19	0.93	1.16
Sulfitobacter mediterraneus	0	0.24	1.22	0.80	1.47	1.41
Epibacterium scottomollicae	1.23	0.62	1.43	1.35	1.13	1.22
Ruegeria conchae	0	0.24	1.55	0.82	1.00	1.04
Ruegeria faecimaris	0.92	0.24	1.07	1.02	0.46	0.27
Octadecabacter temperatus	0	0.44	1.07	0.82	0.46	0.79
Other	65.9	63.8	49.7	49.5	58.5	58.3

Table S6. Summary of the effects observed at different levels of the organisms in *M. galloprovincialis* after 96h exposure to PET-MF. The general effects on functional immune parameters in hemolymph, antioxidant enzyme activities and histopathological changes in gills and digestive gland are reported (from [19]). F1: 10 μg/L, F2: 100 μg/L. * Data on MF accumulation, expressed as % MF/mussel as a function of nominal exposure concentration are also reported.

PET-MF	Hemolymph	Gills	Digestive gland
F1 (10 μg/L)	**Hemocyte stress**: ↓ LMS<br>**Extracellular defenses:**<br>↑ Reactive Oxygen Species<br>↑ Nitric Oxide<br>↑ Lysozyme activity	**Oxidative stress**: ↑ CAT; ↑ GST<br>↑ **Histopathological damage** (cilia disorganization, hemolymphatic vessel enlargement)<br>* **MF accumulation**: 0.07 %	**Inflammation**: Hemocytic infiltration<br>↑ **Histopathological damage:** (increase mucous cell, hypertrophic tubules)<br>* **MF accumulation:** 0.58%
F2 (100 μg/L)	**Hemocyte stress**: ↓ LMS<br>**No induction of extracellular defenses**	**Oxidative stress**: ↑ CAT<br>**Inflammation**: Hemocytic infiltration<br>↑↑ **Histopathological damage** (brown cells)<br>**MF accumulation:** 0.04%	**Oxidative stress**: ↑ CAT; ↑ GST<br>**Inflammation**: Hemocytic infiltration<br>↑↑ **Histopathological damage** (degenerated tubules)<br>* **MF accumulation**: 0.26%
